# Homeobox gene expression in acute myeloid leukemia is linked to typical underlying molecular aberrations

**DOI:** 10.1186/s13045-014-0094-0

**Published:** 2014-12-24

**Authors:** Karolina Skvarova Kramarzova, Karel Fiser, Ester Mejstrikova, Katerina Rejlova, Marketa Zaliova, Maarten Fornerod, Harry A Drabkin, Marry M van den Heuvel-Eibrink, Jan Stary, Jan Trka, Julia Starkova

**Affiliations:** CLIP–Childhood Leukaemia Investigation Prague, Department of Paediatric Hematology and Oncology, 2nd Faculty of Medicine, Charles University Prague, Prague, Czech Republic; CLIP–Childhood Leukaemia Investigation Prague, Department of Paediatric Hematology and Oncology, 2nd Faculty of Medicine, University Hospital Motol, Prague, Czech Republic; Department of Paediatric Hematology and Oncology, 2nd Faculty of Medicine, Charles University Prague and University Hospital Motol, Prague, Czech Republic; Department of Pediatric Oncology and Hematology, ErasmusMC-Sophia Childrens Hospital, Rotterdam, The Netherlands; Department of Hematology and Oncology, Medical University of South Carolina, Charleston, SC USA; Department of Biochemistry, ErasmusMC, Rotterdam, The Netherlands

**Keywords:** Acute myeloid leukemia, Homeobox genes, Myelopoiesis, Epigenetic modifiers

## Abstract

**Background:**

Although distinct patterns of homeobox (*HOX)* gene expression have been described in defined cytogenetic and molecular subsets of patients with acute myeloid leukemia (AML), it is unknown whether these patterns are the direct result of transcriptional alterations or rather represent the differentiation stage of the leukemic cell.

**Method:**

To address this question, we used qPCR to analyze mRNA expression of *HOXA* and *HOXB* genes in bone marrow (BM) samples of 46 patients with AML and sorted subpopulations of healthy BM cells. These various stages of myeloid differentiation represent matched counterparts of morphological subgroups of AML. To further study the transcriptional alterations of *HOX* genes in hematopoiesis, we also analyzed gene expression of epigenetic modifiers in the subpopluations of healthy BM and leukemic cells.

**Results:**

Unsupervised hierarchical clustering divided the AMLs into five clusters characterized by the presence of prevalent molecular genetic aberrations. Notably, the impact of genotype on *HOX* gene expression was significantly more pronounced than that of the differentiation stage of the blasts. This driving role of molecular aberrations was best exemplified by the repressive effect of the *PML-RARa* fusion gene on *HOX* gene expression, regardless of the presence of the *FLT3/ITD* mutation. Furthermore, *HOX* gene expression was positively correlated with mRNA levels of histone demethylases (*JMJD3* and *UTX*) and negatively correlated with gene expression of DNA methyltranferases. No such relationships were observed in subpopulations of healthy BM cells.

**Conclusion:**

Our results demonstrate that specific molecular genetic aberrations, rather than differentiation per se, underlie the observed differences in *HOX* gene expression in AML. Moreover, the observed correlations between epigenetic modifiers and *HOX* ex pression that are specific to malignant hematopoiesis, suggest their potential causal relationships.

**Electronic supplementary material:**

The online version of this article (doi:10.1186/s13045-014-0094-0) contains supplementary material, which is available to authorized users.

## Introduction

The clustered homeobox (*HOX*) genes encode a large family of transcription factors characterized by the presence of a highly conserved nucleotide sequence called the homeodomain. This 61-amino-acid helix-turn-helix domain is responsible for the binding of HOX proteins to their target DNA sequences. In humans, the 39 *HOX* genes are organized into four genomic regions (the *HOXA, B, C* and *D* clusters) located on four chromosomes (chromosomes 7, 17, 12 and 2, respectively). Each cluster consists of 9 – 11 genes arranged in the same orientation and in paralogous groups [1,2].

*HOX* genes play essential roles during embryogenesis by controlling cell fate along the anterior-posterior axis and specifying segment identity [3-5]. The characteristic expression of *HOX* genes can also be detected in various adult tissues [6,7]. During hematopoiesis, the highest expression of *HOX* genes occurs in the stem and early hematopoietic progenitor cells. During maturation, *HOX* expression gradually decreases, and it is minimal in differentiated hematopoietic cells [8,9]. The expression of *HOX* genes throughout the maturation of hematopoietic cells is tightly regulated, suggesting that disruption of this regulation contributes to the process of malignant transformation.

The oncogenic potential of *HOX* genes in leukemia has been intensively studied for more than two decades. Several chromosomal translocations in leukemia involve *HOX* genes either directly (e.g., *NUP98-HOX* fusion) or via their upstream regulators (e.g., MLL rearrangements) [10-13]. Moreover, the overexpression of certain *HOX* genes and their cofactors are known as poor prognostic markers in leukemia patients [14-16]. The overexpression of *HOX* genes is believed to induce myeloproliferation, which together with additional aberrations, may lead to leukemia.

The regulation of gene expression during hematopoiesis is controlled by the cooperation of transcription factors and the dynamic architecture of chromatin. The specific epigenetic landscape influences target gene accessibility. As major executors of epigenetic regulation, chromatin-modifying enzymes mediate DNA and histone modifications responsible for the unique dynamics of chromatin observed throughout hematopoiesis. The deregulation of this process likely contributes to the malignant transformation of hematopoietic cells.

In embryogenesis, spatio-temporal expression of *HOX* genes is regulated by the trithorax-group (TrxG) and polycomb-group (PcG) proteins. *PcG* genes maintain *HOX* gene silencing through methylation of histone 3 lysine 27 (H3K27). In contrast, *TrxG* genes are responsible for maintaining previously established *HOX* gene expression through trimethylation of histone 3 lysine 4 (H3K4) [8,17]. A similar effect of *PcG* and *TrxG* genes has been proposed in the regulation of *HOX* gene expression in hematopoiesis as suggested by the severe defects of hematopoietic cells that have been reported in *PcG* and *TrxG* knock-out models [18,19]. In addition, the H3K4 demethylase *LSD1* and JmjC-domain-containing H3K27 demethylases *JMJD3 (KDM6B)* and *UTX (KDM6A)* have been shown to contribute to *HOX* gene regulation in embryonic development [20,21]. LSD1 establishes an inactive chromatin configuration by H3K4 demethylation, whereas JMJD3 and UTX activate chromatin by demethylation of H3K27. Finally, DNA methylation has been shown to participate in the establishment of *HOX* gene expression patterns, further supporting the role of epigenetics in the regulation of these genes [22].

In this paper, we sought to determine whether the pattern of leukemic *HOX* gene expression was primarily driven by the differentiation stage of hematopoietic cells or determined *de novo* during the process of malignant transformation. To approach this question, the expression patterns of the *HOX* genes were correlated with the molecular genetics and morphological characteristics of the leukemic cells of patients with childhood acute myeloid leukemia (AML). To further study the regulation of *HOX* gene expression, we also examined the relationships of chromatin modifiers and *HOX* genes in normal and malignant myelopoiesis.

## Methods

### BM samples of healthy donors and patients with AML

Subpopulations of healthy BM representing developmental stages of hematopoiesis were sorted from the samples of healthy volunteers or minimal residual disease (MRD)-negative leukemia patients in long-term complete remission (Fluorescence Activated Cell Sorter (FACS) Aria, BD, San Jose, CA, USA). The combination of surface markers that was used to identify the particular stages of myeloid lineage differentiation is listed in Table 1. To ensure adequate analysis sensitivity, we pooled the sorted samples of each subpopulation from five control donors and processed them as described below.

**Table 1:** **Identification of subpopulations of healthy BM cells**

	**Normal sorted populations of myeloid lineage**		**Counterparts with FAB AML**	
	**Granulocyte lineage subpopulations**			
G1	myeloid progenitor	CD117+CD15-CD11b-	AML M1	AML M2
G2	promyelocyte	CD117+CD15+CD11b-	AML M3	AML M2
G3	promyelocyte-myelocyte	CD117-CD13++CD16-		
G4	myelocyte	CD117-CD13dimCD16-		
	**Monocyte lineage subpopulations**			
M1	myeloblast	CD34+SSc++HLA DR+CD33-	AML M1-M0	
M2	myelo/monoblast	CD34+Ssc++HLA DR+CD33+	AML M2	
M3	promonocyte	CD34-FSc and Ssc corresponding to monocytes CD33+CD14-	AML M4 - AML M5a	
M4	monocyte	CD34-FSc and Ssc corresponding to monocytes CD33+CD14+	AML M5b	

Main characteristics of stages of myeloid lineage differentiation – surface markers and their counterparts with morphological subtypes of AML.

In total, 46 patients with childhood AML enrolled in the study were diagnosed and treated from 1998 to 2010 at the Czech Pediatric Hematology Working Group centers (Additional file 1: Table S1). Following the University Hospital Motol ethical committee's approval number P304/12/2214 and written informed consent, mononuclear cells were isolated from the diagnostic BM samples using a density gradient medium (Ficoll-Paque Plus, GE Healthcare Life Sciences, Uppsala, Sweden) and stored at −80°C.

RNA from both the patient samples and the healthy donor BM subpopulations were isolated with RNeasy Mini Kit (Qiagen, Hilden, Germany) and transcribed to cDNA using the iScript kit (Bio-Rad, Hercules, CA, USA).

### Real-time quantitative polymerase chain reaction (qPCR)

The quantification of gene expression was performed using the iCycler iQ System (BioRad, Hercules, CA, USA). The primer design and qPCR conditions for amplification of the *HOXA* and *HOXB* genes in the sorted populations (*HOXA3, A4, A5, A6, A7, A9, A10, B2, B4, B5, B6,* and *B7*) and patient samples (*HOXA1, A3, A4, A5, A6, A7, A9, A10, A11, A13, B1, B2, B4, B5, B6, B7, B8,* and *B9*) as well as the chromatin modifier genes (*PcG* family: *EZH2* and *BMI1; Trx* family: *MLL; DNMTs: DNMT1, DNMT3a,* and *DNMT3b* and histone demethylases: *JMJD3, UTX* and *LSD1*) were performed as previously described [14,15,20,23-25]. To normalize the gene expression levels, we used the *ABL1* gene, which is known to be stably expressed during the development of myeloid lineage cells.

### Mutation analysis

The mutation statuses of the *NPMI, NRAS, KRAS, CEBPa*, *c-KIT* and *FLT3* genes were determined in 12 patients for whom material was available and who were negative for the presence of the four major molecular aberrations (Additional file 1: Table S1). The analysis was performed by qualitative PCR followed by the sequencing of particular amplicons with the primers and PCR conditions as described earlier [26-29].

### Statistical analysis

Data were analyzed using the statistical software packages Prism (GraphPad, La Jolla, CA, USA), Excel (Microsoft Corporation, Redmond, WA, USA), StatView (SAS Institute, Cary, NC, USA) and R-project (Vienna, Austria). The statistical significance of the differences among the subgroups of samples was assessed using non-parametric tests (Mann–Whitney and Kruskal–Wallis tests with Dunn’s multiple comparison post test). Gene expression correlations were estimated by Spearman’s rank correlation. Unsupervised hierarchical cluster analysis (HCA), performed with the Genesis software (Institute for Genomics and Bioinformatics, Graz University of Technology (IGB-TUG), Graz, Austria), was used to identify the subgroups of samples with similar gene expression patterns.

## Results

### Expression patterns of *HOX* genes in sorted subpopulations of healthy BM cells representing different stages of myelopoiesis

Using FACS, we obtained eight subpopulations of normal BM donor cells, based on characteristic surface markers, which represent distinct stages of myeloid differentiation. The subpopulations corresponding with particular maturation stages were selected based on our expertise and previously published studies (Table 1) [30-32]. Notably, we frequently observed the asynchronous expression of antigens and the overlap of immunophenotypic maturation stages in the leukemic blasts.

**Figure 1:**
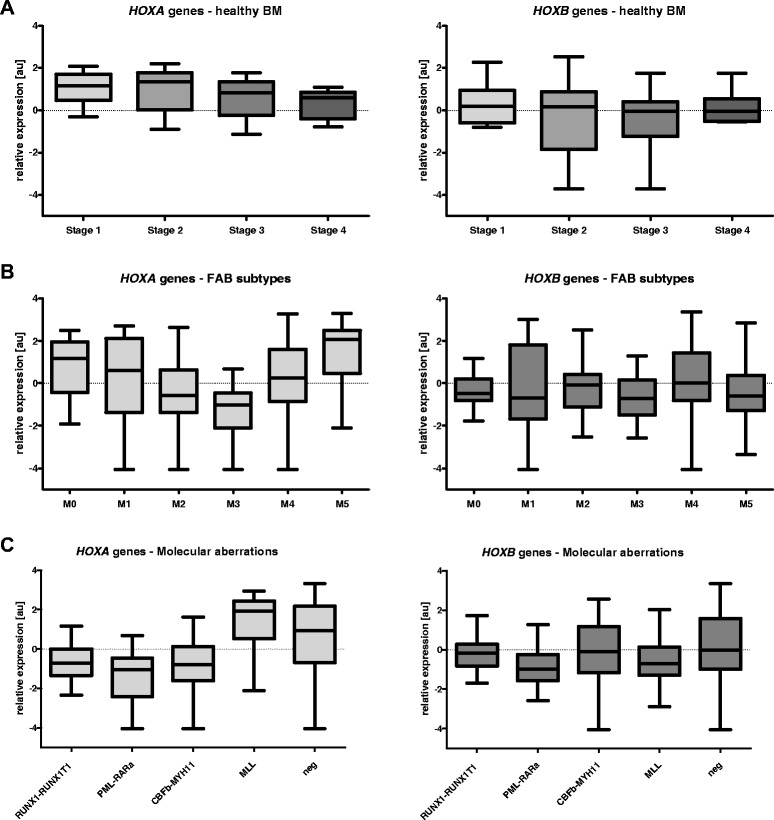
**Expression levels of** ***HOXA*** **and** ***HOXB*** **genes in subpopulations of healthy BM and samples of patients with AML. A**. four consecutive stages of myeloid lineage differentiation; **B**. morphological subgroups of AML patients; **C**. subgroups of AML with typical molecular aberrations.

To better demonstrate the dynamics of *HOX* gene expression throughout hematopoiesis, data from the subpopulations of the two developmental lineages of myelopoises (granulocytic and monocytic) with the parallel differentiation stage were pooled together. This resulted in the discernment of four consecutive stages of myeloid development (stage 1 = G1 + M1, stage 2 = G2 + M2, stage 3 = G3 + M3 and stage 4 = G4 + M4). In accordance with previously published data, the expression of *HOXA* and particular *HOXB* (*HOXB2* and *HOXB4*) genes gradually decreased during myeloid maturation (Figure 1A and Additional file 2: Figure S1). As assessed by comparisons on an one-to-one basis we also observed a clear positive correlation of *HOX* gene expression within *HOXA* cluster and *HOXB* cluster as well as between both clusters (Additional file 3: Figure S2).

### Expression patterns of *HOX* genes in BM samples from patients with childhood AML

Distinctive *HOX* gene expression patterns were observed among the French-American-British classification (FAB) AML subtypes (Kruskal-Wallis test: p < 0.0001 for the *HOXA* and p = 0.0016 for the *HOXB* cluster; Figure 1B and Additional file 4: Figure S3). The M3 FAB subtype had the lowest levels of *HOXA* and *HOXB* gene expression compared with other FAB subtypes (see Additional file 4: Figure S3). In contrast, AML M5 had the highest *HOXA* gene expression levels, along with the largest number of individual *HOXA* genes showing significant differential expression (Additional file 4: Figure S3). Significant differences were also found among subgroups defined according to molecular genetics (i.e., Kruskal-Wallis test: p < 0.0001 for the *HOXA* and p = 0.0001 for the *HOXB* cluster; Figure 1C and Additional file 5: Figure S4). Patients with *PML-RARa* fusion showed the lowest levels of *HOXA* and *HOXB* gene expression, while those with *MLL* rearrangements expressed *HOXA* genes at the highest levels (the majority of individual *HOX* gene comparisons revealed significant differential expression in the *PML-RARa* and *MLL*+ patients, respectively). Moreover, unsupervised hierarchical clustering based on *HOX* expression divided the leukemias into five main clusters characterized by the presence or absence of prevalent gene rearrangements, i.e., *PML-RARa*, *RUNX1-RUNX1T1(AML1-ETO)*, *CBFb-MYH11* and *MLL* alterations (Additional file 6: Figure S5). Interestingly, three patients from cluster 1 (i.e., those having the overall highest levels of *HOX* gene expression and absence of these translocations) harbored a mutation in the *NPM1* gene, similar to what has been reported in adult AML [16].

High risk (HR) patients expressed *HOXA* genes at significantly higher levels compared with patients who were assigned to the standard risk (SR) group (p < 0.0001 for *HOXA3 - A13* and p = 0.0004 for *HOXA1*). However, no differences were observed in *HOXB* expression between high and low risk patients. In addition, both *HOXA* and *HOXB* gene expression were not found to be related to the patient age or risk stratification (Additional file 7: Figure S6; risk group stratification of childhood AML (AML-BFM 98 and 2004): standard risk - FAB M1/M2 with Auer rods, M3, M4eo, Down sy, t(8;21), t(15;17), inv(16), and ≤ 5% of blasts in BM at D15 (except M3); high risk - others).

### Impact of molecular aberrations on *HOX* gene expression within the morphological subgroups of AML

**Figure 2:**
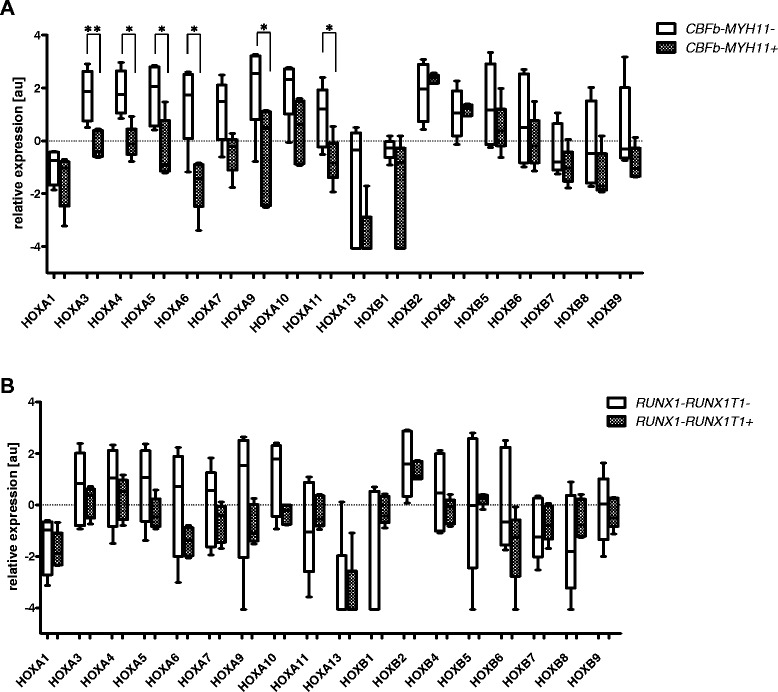
**Differing** ***HOX*** **gene expression levels observed in FAB subtypes with respect to the presence of typical molecular aberrations. A**. *CBFb-MYH11*+/− patients with AML M4. Asterisks indicated statistical significance (* ≤ 0.05, ** ≤ 0.01; additional borderline significance: p = 0.0679 for *HOXA7, HOXA10* and *HOXB9*, p = 0.0732 for *HOXA13*); **B**. *RUNX1-RUNX1T1*+/− patients with AML M2. Asterisks indicated statistical significance (* ≤ 0.05, ** ≤ 0.01; additional borderline significance: p = 0.1745 for *HOXA5*, p = 0.1745 for *HOXA9* and P = 0.1172 for *HOXA10*).

The effects of genetic aberrations on *HOX* gene expression were even more apparent when analyzed within the morphological FAB subtypes. In AML M4, the *CBFb-MYH11*+ patients exhibited statistically significant levels of lower *HOX* expression compared with those lacking the rearrangement. For AML M2, the *RUNX1-RUNX1T1+* patients tended to show reduced levels of *HOX* gene expression compared with patients without the rearrangement (Figure 2A and 2B; p values indicated in figure legend).

### Different effect of *FLT3/ITD* on *HOX* gene expression in the presence of PML-RARa fusion gene

**Figure 3:**
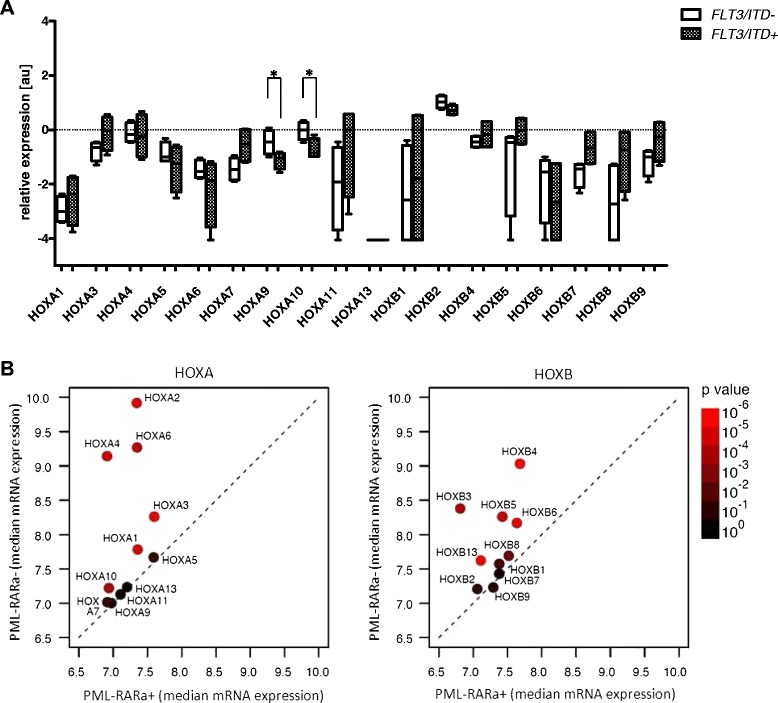
**Differing** ***HOX*** **gene expression levels observed in** ***PML-RARa*** **+ patients with respect to the presence of** ***FLT3/ITD*** **. A**. *HOX* gene expression in the subgroup of *PML-RARa*+ patients with *FLT3/ITD* compared to the other *PML-RARa+* patients. Asterisks indicated statistical significance (* ≤ 0.05, ** ≤ 0.01; additional borderline significance: p = 0.0833 for *HOXA7*); **B**. *HOX* gene expression levels in *FLT3/ITD+* patients with *PML-RARa* compared to the other *FLT3*/*ITD* patients. Median expression (log2) of *HOXA* (left) or *HOXB* genes is indicated by bullets colored according to p values (Mann–Whitney tests) of differences between *PML-RARa* + and other groups. Color legend indicated at right. Dashed line indicating equal expression is also provided.

In adults with normal cytogenetic AML, *NPMI1* mutations are associated with high *HOX* expression and those leukemias have a higher frequency of *FLT3* mutations [33]. In our samples, *HOX* gene expression in the *PML-RARa*+ patients remained at very low levels regardless of the presence of *FLT3/ITD* mutations (*FLT3/ITD*+ (N = 4) vs. *FLT3/ITD*- (N = 4); Figure 3A; p values indicated in figure legend). In contrast, in the absence of a *PML-RARa* fusion, *HOX* levels were higher when *FLT3* was mutated. These results were further emphasized by the analysis of gene expression data from a larger (N = 48) independent cohort of *FLT3/ITD+* childhood AML patients [34], which demonstrated that *HOXA* and *HOXB* gene levels were significantly reduced in *FLT3/ITD*+ patients with *PML-RARa* (N = 12) compared to those without the fusion gene (N = 36; Figure 3B).

### *HOX* gene expression patterns in corresponding differentiation stages of normal and malignant myelopoiesis

**Figure 4:**
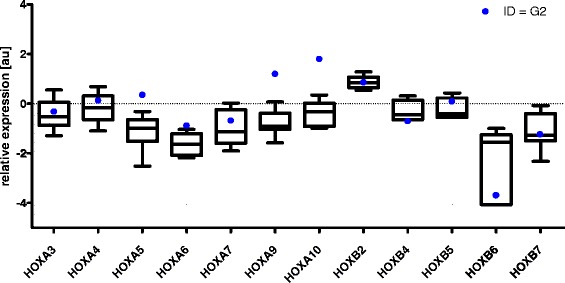
***HOX*** **gene expression patterns in corresponding differentiation stages of normal and malignat hematopoiesis.** Comparison of *HOX* gene expression patterns between AML M3 and sorted subpopulation of healthy BM cells (ID = G2).

As indicated above, we sorted the subpopulations of normal BM cells from the healthy donors according to the specific stage of myelopoiesis. Our gating strategy enabled a comparison of these subpopulations with the FAB subtypes of AML patients exhibiting the similar stage of myeloid maturation arrest. The list of AML subtypes assigned to particular stages of myelopoiesis is provided in Table 1. Differential *HOX* gene expression patterns were identified between the normal and malignant hematopoietic counterparts, as demonstrated by comparing AML M3 with the corresponding stage of promyelocytes (ID = G2). These differences were particularly evident for *HOXA5, HOXA6, HOXA9, HOXA10* and *HOXB4* (Figure 4). Similarly, differential expression of *HOXA3, HOXA4, HOXA5, HOXA6, HOXA7, HOXA9*, *HOXA10, HOXB5* and *HOXB6* distinguished leukemic cells of the M5a and M5b FAB subtypes from the matched normal counterparts, represented by the sorted M3 and M4 population, respectively (Additional file 8: Figure S7A and 7B).

### Expression patterns of chromatin modifiers and their role in *HOX* gene regulation

**Figure 5:**
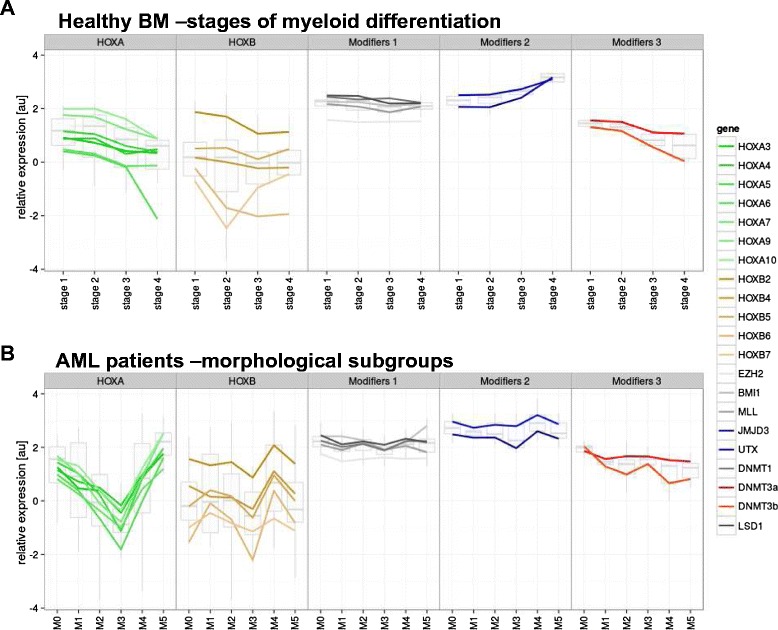
**Expression patterns of chromatin modifiers and** ***HOX*** **genes in subpopulations of healthy BM and AML cells. A**. Subpopulations of healthy BM; **B**. AML patient subgroups. Lines connect values between categories (e.g., differentiation stages) to visually enhance depiction of trend across subgroups.

In subpopulations of healthy BM cells, we analyzed the expression of chromatin modifying genes, previously shown to contribute to *HOXA* and *HOXB* regulation during embryogenesis [21,35,36]. Based on observed expression in the sorted subpopulations, the chromatin modifiers were divided into three groups. The first group, “Modifiers 1”, included genes that did not exhibit varying expression levels during differentiation (*EZH2*, *BMI1*, *MLL, LSD1* and *DNMT1*). The second group, “Modifiers 2”, consisted of genes showing increased expression during hematopoiesis (*JMJD3* and *UTX*). Expression levels of the third group, “Modifiers 3” (*DNMT3a* and *DNMT3b*), showed a decrease concomitant with differentiation, which were statistically inversely correlated with Modifiers 2 (R = −0.922; Figure 5A). However, we did not observe a clear pattern of corresponding *HOX* gene expression changes in these cells.

**Figure 6:**
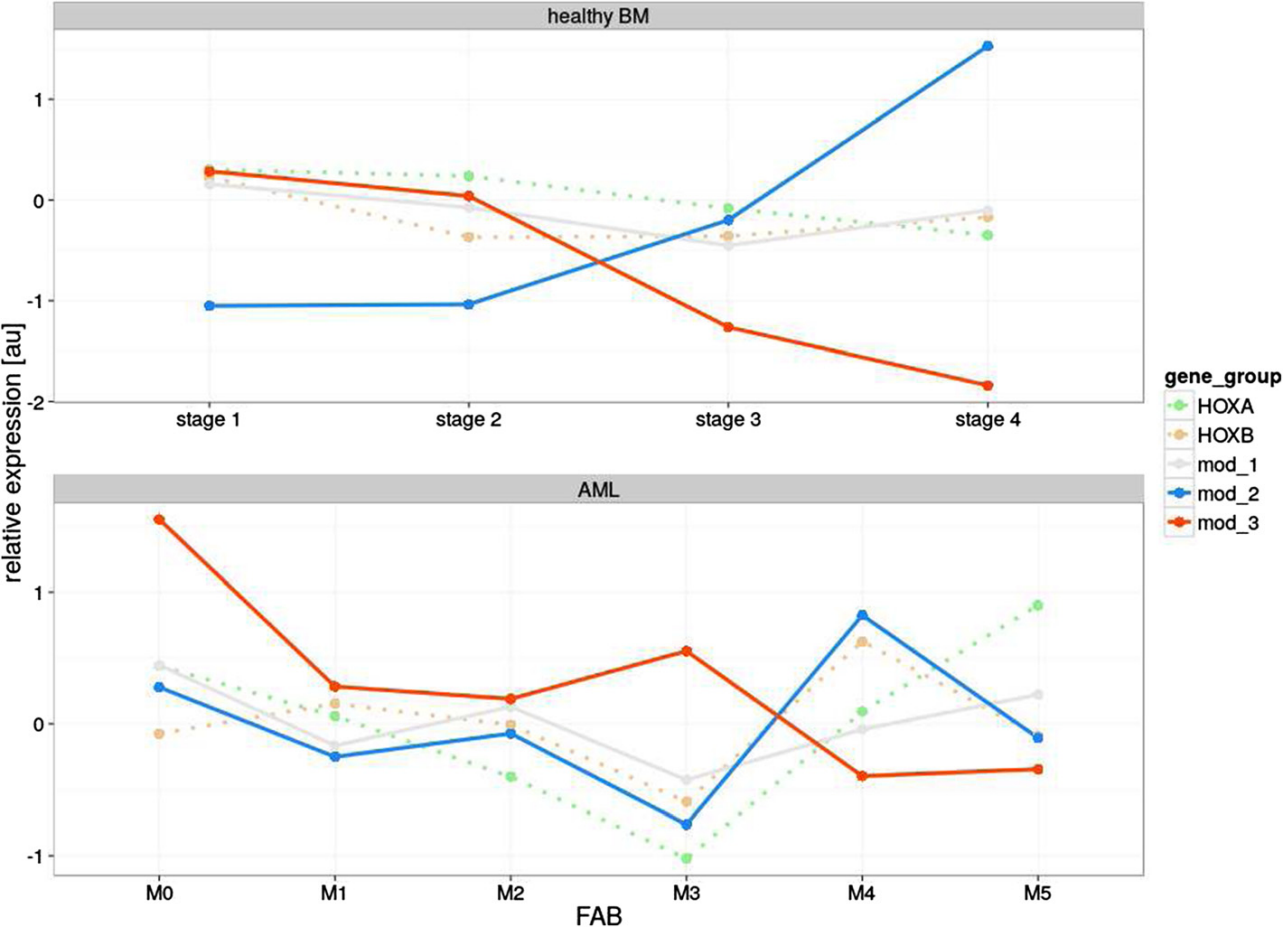
**Relationship of expression patterns of chromatin modifiers and** ***HOX*** **genes in subpopulations of healthy BM and AML cells.** Lines connect values between categories (e.g., differentiation stages) to visually enhance depiction of trend across subgroups.

In leukemic cells, the expression of Modifier 1 genes, except for *BMI1*, paralleled their normal counterparts, being largely unchanged among the morphological AML subgroups. The expression of Modifier 2 and 3 genes markedly differed among AML samples (Figure 5B). In contrast to normal cells, the expression of Modifier 2 and 3 genes appeared to mirror the differences in *HOX* mRNA levels. In general, *HOXB* expression was positively correlated with Modifier 2 genes (e.g., H3K27 demethylases; R = 0.874). The correlation of *HOXA* gene expression levels with Modifiers 2 genes was less pronounced (R = 0.506) in all cases with the exception of the AML M3 subgroup. For the Modifier 3 genes (e.g., *DNMTs*), there was a substantial negative correlation with *HOXB* expression (R = −0.442; Figure 6). However, in contrast to normal hematopoiesis, the observed inverse correlation between Modifier 2 and 3 genes was much less pronounced (except for AML M3 and M4; R = −0.178; Figure 6).

For individual genes in the leukemic samples (Additional file 9: Figure S8), there were several notable correlations. For instance, the expression of *JMJD3* with *HOXB4* and *HOXB6* was strongly positively correlated (p = 0.0003 and 0.0012, respectively), while negatively correlated with *DNMT3b* (p = 0.03). There was also strong correlation on a one-to-one basis between genes in the *HOXA* cluster and for genes in the *HOXB* cluster. However, in contrast with the healthy samples, there was only a weak correlation between the particular *HOX* genes from different *HOX* clusters.

## Discussion

Several reports have demonstrated that *HOX* genes are not only potent regulators of embryonic development but also play significant roles in the regulation of many processes in adult organisms, including hematopoiesis [37-39]. The overall role of *HOX* clusters in addition to that of particular *HOX* genes in hematopoiesis have been revealed by various knock-out and overexpression studies of human hematopoietic cells or by studies using mouse models [40-43]. However, the degree to which *HOX* genes contribute to the process of leukemogenesis has not yet been elucidated.

The aberrant expression of *HOX* genes has been reported in the majority of leukemia patients. However, it remains unknown whether this aberrant expression represents a genuine driver of leukemogenesis or a passenger effect reflecting a differentiation block. Another possible explanation takes into consideration an impact of the molecular aberrations present in leukemic cells with further biological consequences. Here, we attempted to shed light on the expression of *HOX* genes in normal hematopoietic precursor cells versus their malignant counterparts with respect to their differentiation stage arrest in AML.

The crucial prerequisite for such an analysis is the appropriate identification of subpopulations of healthy BM cells representing the stages of myelopoiesis that can be matched to their respective morphological counterparts in AML. We managed to sort these subpopulations and analyzed their *HOX* gene expression patterns. The expression of *HOX* genes was higher at the initial stages of hematopoiesis and gradually decreased with the maturation of the hematopoietic cells, supporting the generally accepted hypothesis that *HOX* genes are strong regulators of hematopoiesis (particularly at the early stages) [37].

A comparison of matched normal and malignant hematopoietic precursor cells at the same differentiation stage demonstrated the distinct expression patterns of the *HOX* genes in the leukemic cells. This indicates that the aberrant patterns of *HOX* gene expression cannot be simply explained by the differentiation statuses at which the cells have been arrested. This is similar to what we previously observed in pediatric patients with ALL, who were found to exhibit differential *HOX* gene expression between the subgroups and their matched normal precursors according to differentiation stage [15]. Altogether, our results support the hypothesis that the dysregulation of *HOX* genes is involved in the process of neoplastic transformation.

The analysis of childhood AML patients revealed a different expression profile of *HOX* genes among the FAB subtypes and the subgroups of patients bearing unique molecular rearrangements. The most diverse subgroup of AML was AML M3, which showed the lowest levels of *HOX* gene expression. This subgroup is characterized by the presence of the *PML-RARa* fusion gene, which generates an aberrant retinoic acid receptor unresponsive to the physiological levels of this molecule. *RUNX1-RUNX1T1+*, *CBFb-MYH11+* and *MLL-*rearranged AML patients also showed unique *HOX* gene expression patterns. *MLL* rearrangements have been previously shown to have a determinant role on *HOX* gene expression [44]. Moreover, we revealed that AML patients bearing the *PML-RARa* fusion gene had low expression levels of the *HOX* genes regardless of *FLT3/ITD* status. This finding is even more interesting considering that *FLT3/ITD* has been shown to be associated with the upregulated expression of *HOX* genes in leukemia patients [33]. Therefore, we performed an analysis of a larger cohort of AML patients [34] from Erasmus MC-Sophia Children’s Hospital and replicated the results drawn from our cohort of pediatric AML patients. This analysis showed that despite the overall upregulation of the *HOX* genes in *FLT3/ITD*+ AML patients, *HOX* gene expression in *FLT3/ITD+ PML-RARa+* patients was significantly lower compared to the *FLT3/ITD+* patients without this fusion protein. Therefore, in this case, the *PML-RARa* fusion gene may be superior to *FLT3/ITD* with respect to its role in the process of malignant transformation. Based on these results, we suggest that AML-specific fusion oncoproteins may impact the upstream pathways that deregulate the *HOX* genes, thereby acting as the major underlying factors of their characteristic expression patterns observed in leukemic cells.

Our analysis of the AML patients also showed significantly lower expression levels of *HOXA* in the SR compared with the HR patients (in accordance with a previous study [14]). These results suggest that the assessment of *HOX* gene expression patterns may allow for the prediction of aggressive cases of leukemia and may therefore be taken into consideration in risk stratification. However, we suggest that this observation is a consequence of the allocation of patients with different molecular aberrations to particular AML risk groups (i.e., *PML-RARa*+ patients with the lowest *HOXA* gene expression levels being assigned to the SR group and *MLL*+ cases with the highest expression levels of *HOXA* genes being allocated to the HR group) and not an independent prognostic factor.

Considering the profound contribution of chromatin modifiers to the embryonic regulation of *HOX* genes and the essential roles of *HOX* genes in hematopoiesis, the dysregulation of chromatin modifiers may deregulate the entire process of hematopoiesis and subsequently lead to malignant transformation. However, the exact roles of epigenetic modifications in the regulation of leukemic *HOX* gene expression remain to be elucidated. It has recently been shown that *HOX* genes possess unique chromatin regions called bivalent domains. These domains are characterized by the presence of both repressive (methylated H3K27) and activating (methylated H3K4) histone methylation marks and are found in genes poised to be activated according to cell-specific requirements [45]. To determine the role of chromatin modifiers in the regulation of *HOX* genes in normal hematopoiesis and leukemogenesis, we analyzed the expression patterns of DNA methyltransferases, histone H3K27/H3K4 demethylases, and selected *PcG* and *TrxG* genes in subpopulations of healthy BM cells and BM samples of patients with AML. We found an inverse correlation of histone demethylase (Modifiers 2) and *DNMT* (Modifiers 3) gene expression in normal and malignant hematopoiesis. In contrast to healthy hematopoiesis, we found an interesting correlation between chromatin modifier gene expression and that of the *HOX* genes in the AML samples. The most pronounced correlation was observed with the AML M3 subtype. The specific relationship of the *HOX* genes with the epigenetic modifiers in this morphological subgroup could be affected by the presence of the *PML-RARa* fusion gene. In particular, *HOX* gene expression was positively associated with the histone H3K27 demethylases, *JMJD3* and *UTX,* and inversely correlated with *DNMT3b*. Notably, both *JMJD3* and *UTX* have recently been suggested to play roles in hematopoiesis [24,46]. Moreover, *UTX* has been shown to directly bind to the *HOXB1* locus [21,24]. Taken together, the results implicate chromatin modifiers in the establishment of the aberrant leukemic expression of *HOX* genes in pediatric AML patients.

Although the expression of *BMI1* was not altered during hematopoiesis, a Spearman correlation analysis showed that this gene was positively correlated with *HOX* gene expression in the leukemic samples. It has been reported that *BMI1* determines the proliferating abilities of the cells by inhibiting the *p16* gene. *HOXA9* was also shown to target *p16* and impair the senescence of cells [47]. Thus, the expression levels of the histone methyltransferase *BMI1* are likely to reflect the proliferation statuses of leukemic cells without directly impacting *HOX* gene expression [48].

Interestingly, the *PML-RARa* and *RUNX1-RUNX1T1* fusion oncogenes have been shown to cooperate with repressive complexes, leading to alterations in chromatin architecture. *PML-RARa* causes profound changes in the epigenetic landscape, mainly by recruiting chromatin-modifying enzymes to target sequences or by the deregulation of their functions [49,50]. Furthermore, recent studies have shown that the degradation of the PML-RARa oncoprotein results in dramatic changes to the landscape of histone modifications [51]. Similarly, *RUNX1-RUNX1T1* has also been shown to recruit epigenetic modifiers to target sequences [52]. These findings together with our data suggest that AML-specific oncoproteins regulate *HOX* gene expression through epigenetic modifications. However, further studies are needed to understand the roles of epigenetic modifiers in the regulation of normal as well as leukemic *HOX* gene expression and their cooperation with AML fusion oncoproteins.

In summary, we found that the expression patterns of the *HOX* genes in leukemic cells are not solely determined by their particular differentiation stages. Conversely, we assume that the specific molecular aberrations that are typical of AML are the major determinants of the leukemic expression patterns of the *HOX* genes. Our results also demonstrate the differing contributions of epigenetic modifiers to *HOX* gene expression in healthy and malignant hematopoiesis.

## Additional files

Additional file 1: Table S1. Patients’ characteristics. Additional file 2: Figure S1. mRNA expression of particular *HOXA* and *HOXB* genes in subpopulations of healthy BM. Additional file 3: Figure S2. Correlation of *HOXA*, *HOXB* and chromatin modifier gene expression in subpopulations of healthy BM. Additional file 4: Figure S3. mRNA expression of particular *HOXA* and *HOXB* genes in morphological subgroups of AML patients. Additional file 5: Figure S4. mRNA expression of particular *HOXA* and *HOXB* genes in subgroups of AML patients defined according to molecular genetics. Additional file 6: Figure S5. Unsupervised HCA of AML patients based on the epxression pattern of *HOX* genes. Additional file 7: Figure S6. Comparison of individual *HOX* gene expression of SR and HR groups. Additional file 8: Figure S7. Comparison of *HOX* gene expression pattern between AML M5 patients and sorted subpopulation of healthy BM (A. AML M5a vs. ID = M3; B. AML M5b vs. ID = M4). Additional file 9: Figure S8. Correlation of *HOX* and chromatin modifier gene expression in AML patients.
