# Supplementary material for: Homeobox gene expression in acute myeloid leukemia is linked to typical underlying molecular aberrations
Source: J Hematol Oncol. 2014 Dec 24;7:94. doi: 10.1186/s13045-014-0094-0 (PMC4310032; doi:10.1186/s13045-014-0094-0)
Supplement: Additional file 1: Table S1. — Patients’ characteristics. [file 13045_2014_94_MOESM1_ESM.doc]

**Additional file 1: Table S1.** Patients’ characteristics

|  | N | % |
| --- | --- | --- |
| **AML patients** | 46 |  |
| M/F | 20/26 | 43/57 |
| Age - median (range) [years] | 10 |  |
| **FAB Subtypes:** | | |
| M0 | 4 | 9 |
| M1 | 3 | 7 |
| M2 | 10 | 22 |
| M3 | 8 | 17 |
| M4 | 11 | 24 |
| M5 | 10 | 22 |
| **Cytogenetics/Molecular genetics:** | | |
| t(15;17) *PML-RARa* | 8 | 17 |
| t(8;21) *RUNX1-RUNX1T1* | 6 | 13 |
| inv(16) *CBFb-MYH11* | 5 | 11 |
| t(11q23) *MLL* rearrangements | 9 | 20 |
| negative^1,2^ | 18 | 39 |
| NK-AML | 8 | 17 |
| *FLT3/ITD*^3^ | 6 | 13 |
| *NPMI1* (mut) | 3 | 6 |
| *CEBPa* (mut) | 0 | 0 |
| *NRAS* (mut) | 0 | 0 |
| *KRAS* (mut) | 0 | 0 |
| *c-KIT* (mut) | 0 | 0 |
| **Risk groups^4^:** | | |
| Standard | 22 | 48 |
| High | 24 | 52 |
| Follow-up - median (range) [months] | 60 |  |
| Relapse | 15 | 33 |
| Died | 16 | 35 |

^1^patients negative for the presence of four chromosomal translocations typical of AML. Data from 3 patients were not available

^2^except of *FLT3/ITD*, mutation status of the analyzed genes was determined in 12 AML pateints from the cluster of negative patients

^3^including 1 patient with point mutation in *FLT3* gene

^4^risk group stratification of childhood AML (AML-BFM 98 and 2004): standard risk - FAB M1/M2 with Auer rods, M3, M4eo, Down sy, t(8;21), t(15;17), inv(16), and ≤ 5% of blasts in BM at D15 (except M3); high risk - others.
