# Supplementary material for: Homeobox gene expression in acute myeloid leukemia is linked to typical underlying molecular aberrations
Source: J Hematol Oncol. 2014 Dec 24;7:94. doi: 10.1186/s13045-014-0094-0 (PMC4310032; doi:10.1186/s13045-014-0094-0)

**Additional file 2: Figure S1.** mRNA expression of particular *HOXA* and *HOXB* genes in subpopulations of healthy BM


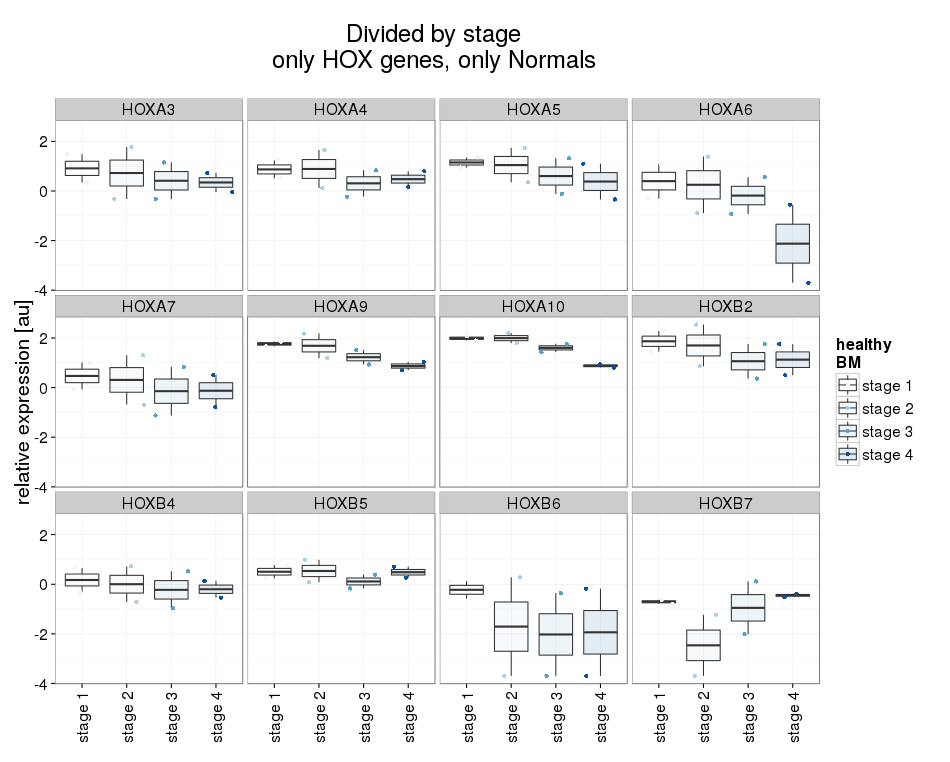

Supplement: Additional file 2: Figure S1. — mRNA expression of particular HOXA and HOXB genes in subpopulations of healthy BM. [file 13045_2014_94_MOESM2_ESM.doc]
