# Supplementary material for: Homeobox gene expression in acute myeloid leukemia is linked to typical underlying molecular aberrations
Source: J Hematol Oncol. 2014 Dec 24;7:94. doi: 10.1186/s13045-014-0094-0 (PMC4310032; doi:10.1186/s13045-014-0094-0)

**Additional file 3: Figure S2.** Correlation of *HOXA*, *HOXB* and chromatin modifier gene expression in subpopulations of healthy BM


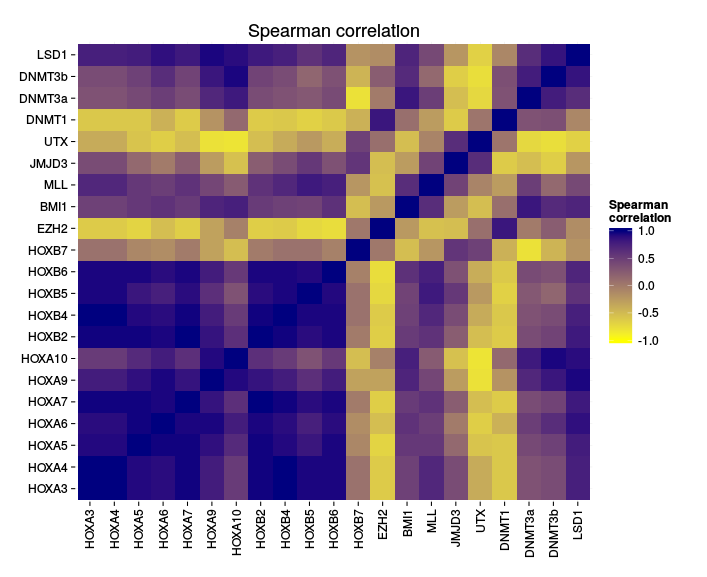

Supplement: Additional file 3: Figure S2. — Correlation of HOXA, HOXB and chromatin modifier gene expression in subpopulations of healthy BM. [file 13045_2014_94_MOESM3_ESM.doc]
