# Supplementary material for: Homeobox gene expression in acute myeloid leukemia is linked to typical underlying molecular aberrations
Source: J Hematol Oncol. 2014 Dec 24;7:94. doi: 10.1186/s13045-014-0094-0 (PMC4310032; doi:10.1186/s13045-014-0094-0)
Supplement: Additional file 4: Figure S3. — mRNA expression of particular HOXA and HOXB genes in morphological subgroups of AML patients. [file 13045_2014_94_MOESM4_ESM.doc]

**Additional file 4: Figure S3.** mRNA expression of particular *HOXA* and *HOXB* genes in morphological subgroups of AML patients

**
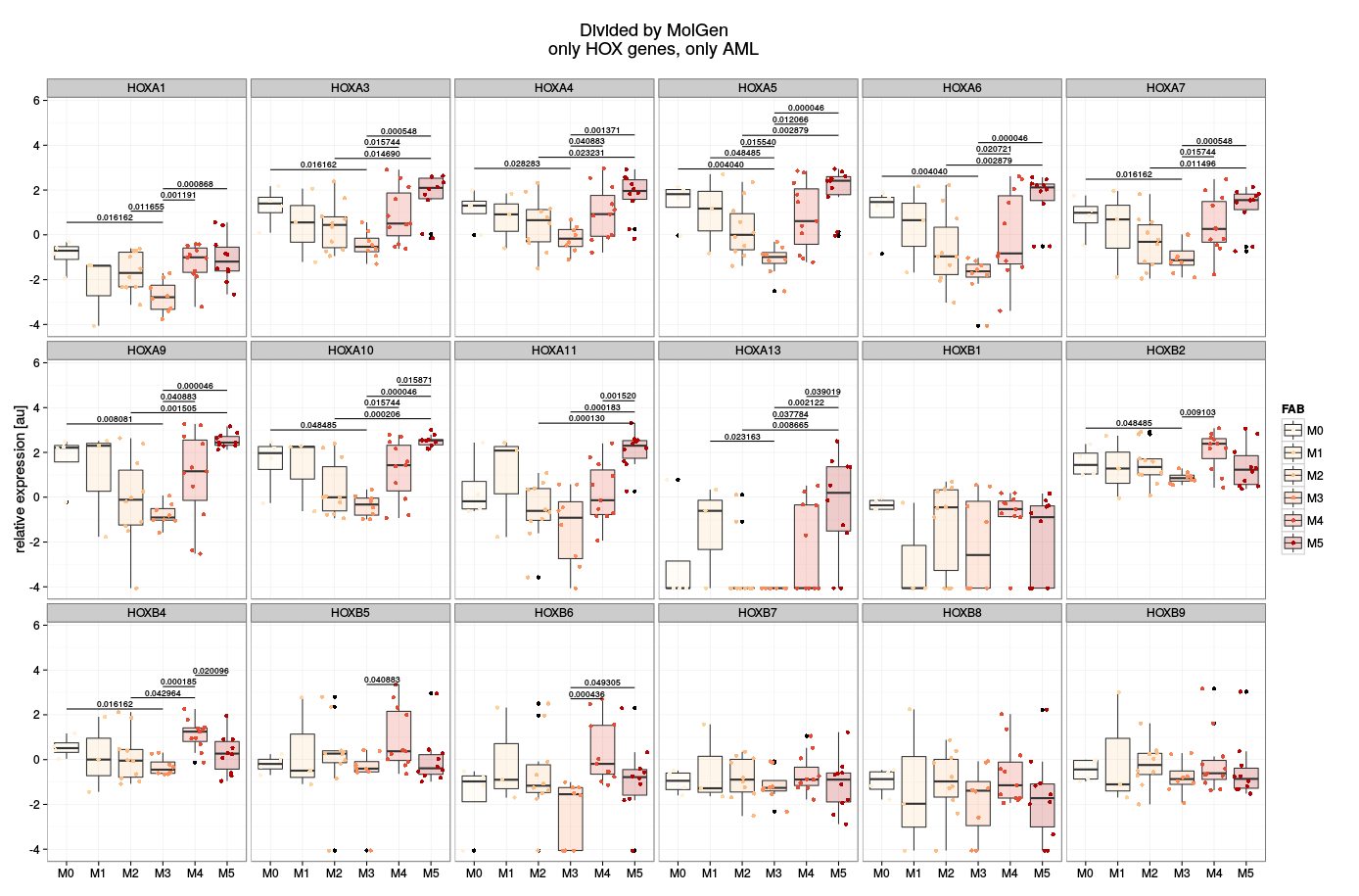
**
