# Supplementary material for: Homeobox gene expression in acute myeloid leukemia is linked to typical underlying molecular aberrations
Source: J Hematol Oncol. 2014 Dec 24;7:94. doi: 10.1186/s13045-014-0094-0 (PMC4310032; doi:10.1186/s13045-014-0094-0)

**Additional file 5: Figure S4.** mRNA expression of particular *HOXA* and *HOXB* genes in subgroups of AML patients defined according to molecular genetics
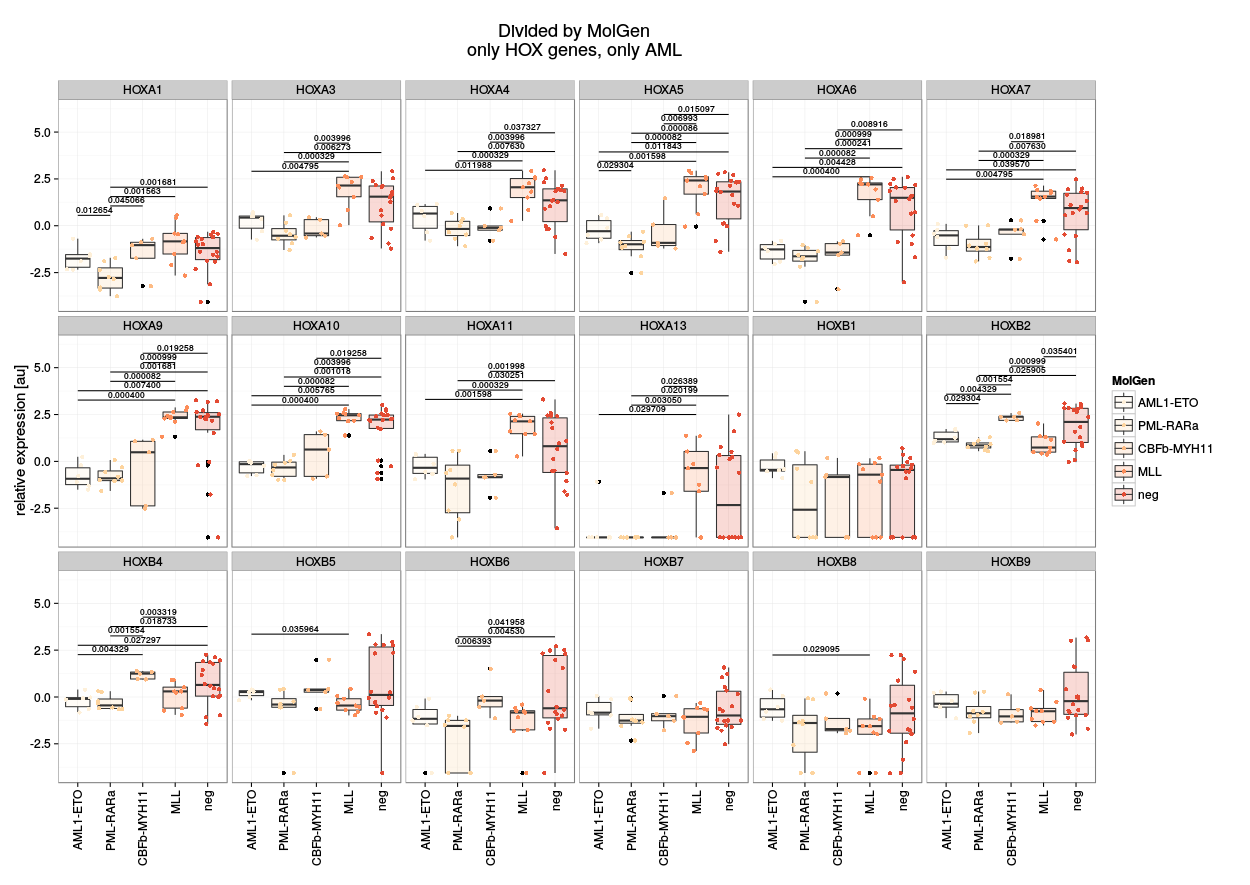

Supplement: Additional file 5: Figure S4. — mRNA expression of particular HOXA and HOXB genes in subgroups of AML patients defined according to molecular genetics. [file 13045_2014_94_MOESM5_ESM.doc]
