# Supplementary material for: Homeobox gene expression in acute myeloid leukemia is linked to typical underlying molecular aberrations
Source: J Hematol Oncol. 2014 Dec 24;7:94. doi: 10.1186/s13045-014-0094-0 (PMC4310032; doi:10.1186/s13045-014-0094-0)

**Additional file 6: Figure S5.** Unsupervised HCA of AML patients based on the epxression pattern of *HOX* genes


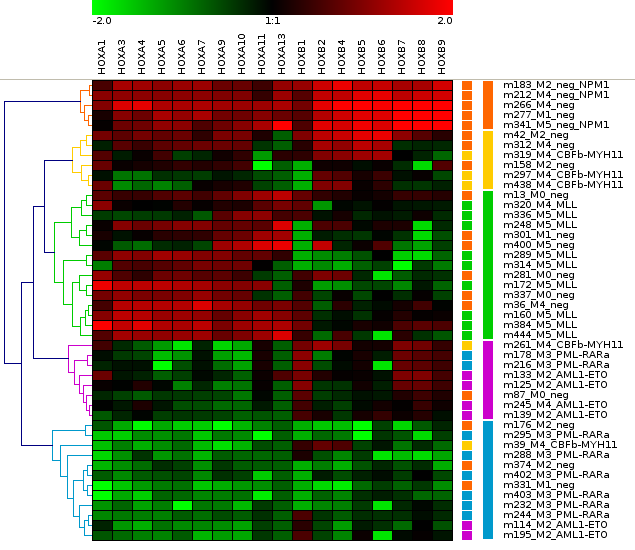

Supplement: Additional file 6: Figure S5. — Unsupervised HCA of AML patients based on the epxression pattern of HOX genes. [file 13045_2014_94_MOESM6_ESM.doc]
