# Supplementary figures and images for: Homeobox gene expression in acute myeloid leukemia is linked to typical underlying molecular aberrations
Source: J Hematol Oncol. 2014 Dec 24;7:94. doi: 10.1186/s13045-014-0094-0 (PMC4310032; doi:10.1186/s13045-014-0094-0)

**Additional file 7: Figure S6.** Comparison of individual *HOX* gene expression of SR and HR groups

**
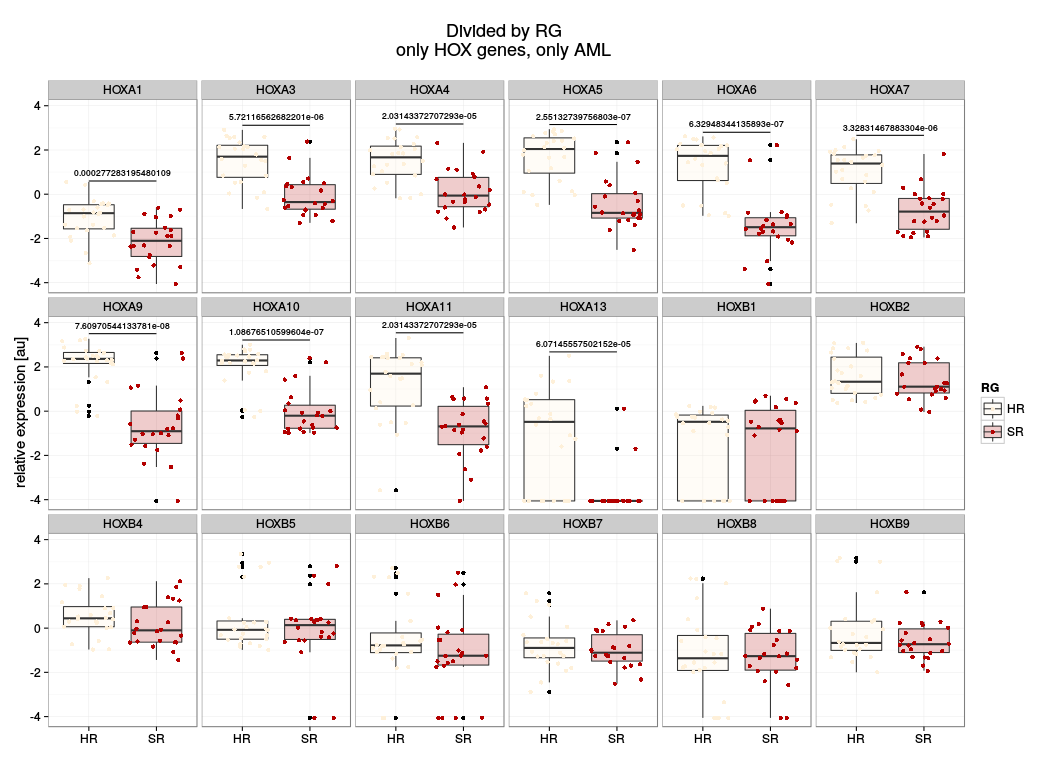
**

Supplement: Additional file 7: Figure S6. — Comparison of individual HOX gene expression of SR and HR groups. [file 13045_2014_94_MOESM7_ESM.doc]
