# Supplementary material for: Homeobox gene expression in acute myeloid leukemia is linked to typical underlying molecular aberrations
Source: J Hematol Oncol. 2014 Dec 24;7:94. doi: 10.1186/s13045-014-0094-0 (PMC4310032; doi:10.1186/s13045-014-0094-0)

**Additional file 8: Figure S7.** Comparison of *HOX* gene expression pattern between AML M5 patients and sorted subpopulation of healthy BM (**A**. AML M5a vs. ID = M3; **B**. AML M5b vs. ID = M4)

**A**
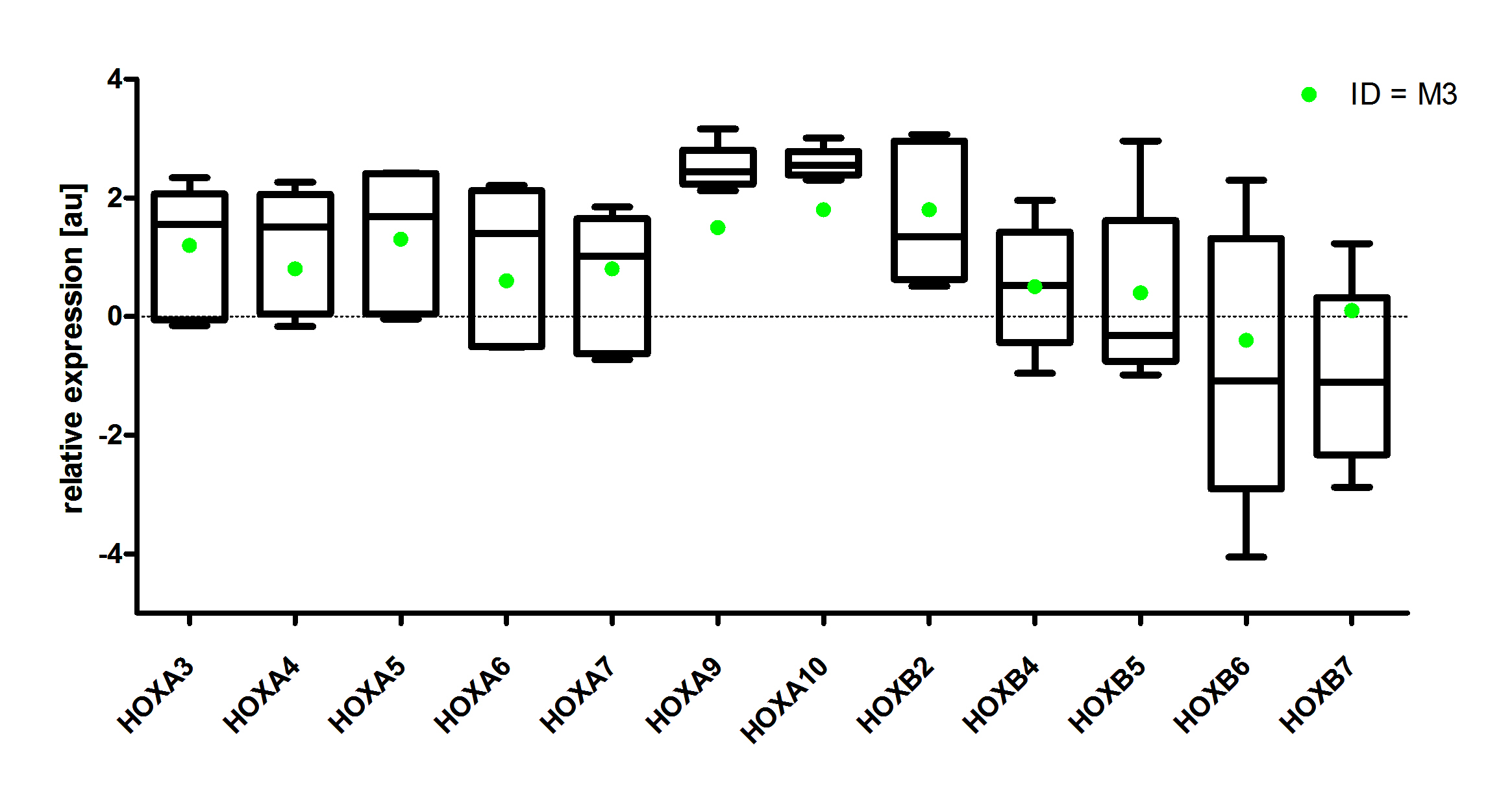


**B**
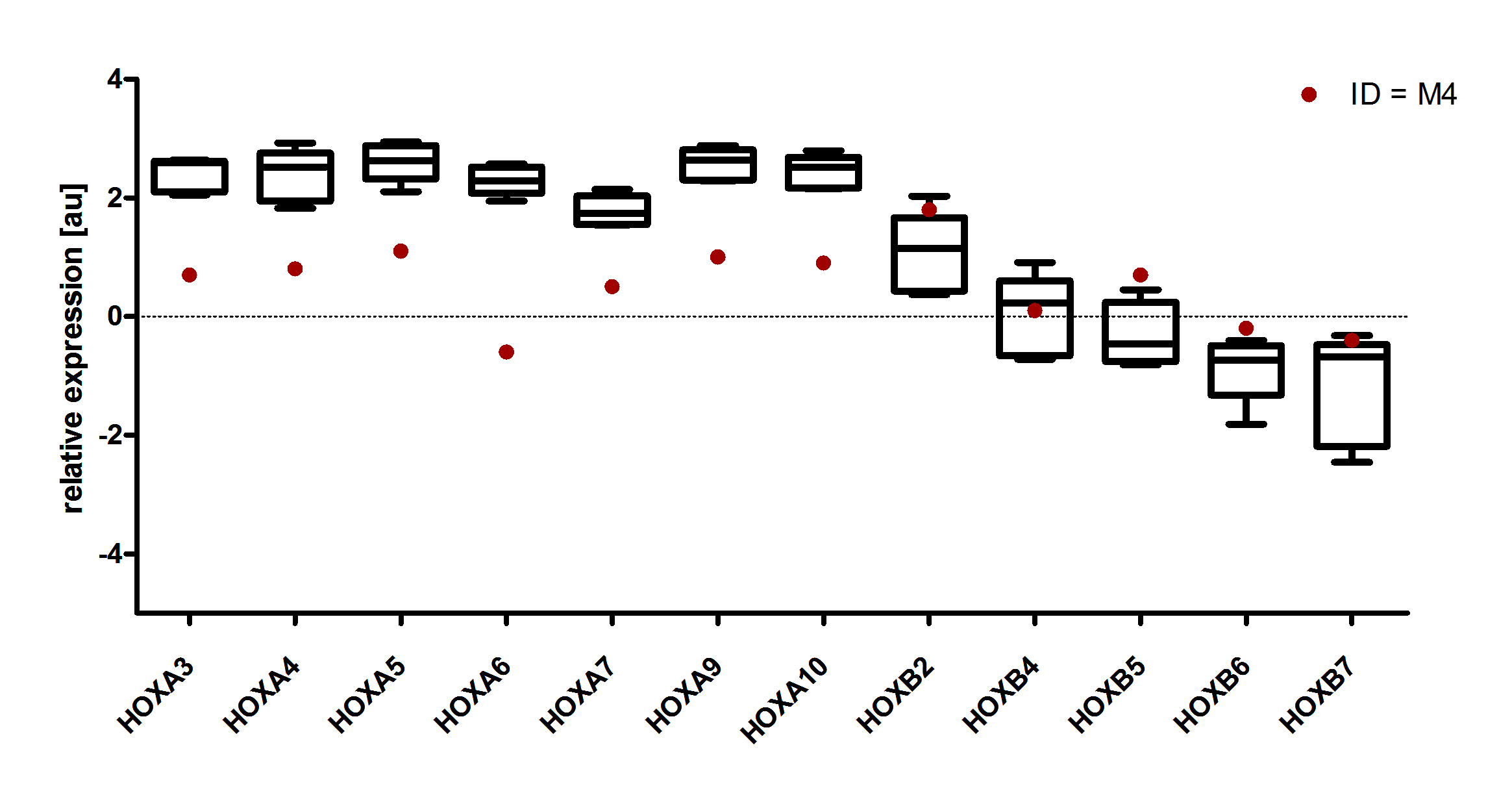

Supplement: Additional file 8: Figure S7. — Comparison of HOX gene expression pattern between AML M5 patients and sorted subpopulation of healthy BM (A. AML M5a vs. ID = M3; B. AML M5b vs. ID = M4). [file 13045_2014_94_MOESM8_ESM.doc]
